# Supplementary material for: Design, Synthesis and Biological Evaluation of Diosgenin-Amino Acid Derivatives with Dual Functions of Neuroprotection and Angiogenesis
Source: Molecules. 2019 Nov 7;24(22):4025. doi: 10.3390/molecules24224025 (PMC6891328; doi:10.3390/molecules24224025)

Supplementary Material for compounds:

DG-1

^{1}H NMR spectra of Compound DG-1

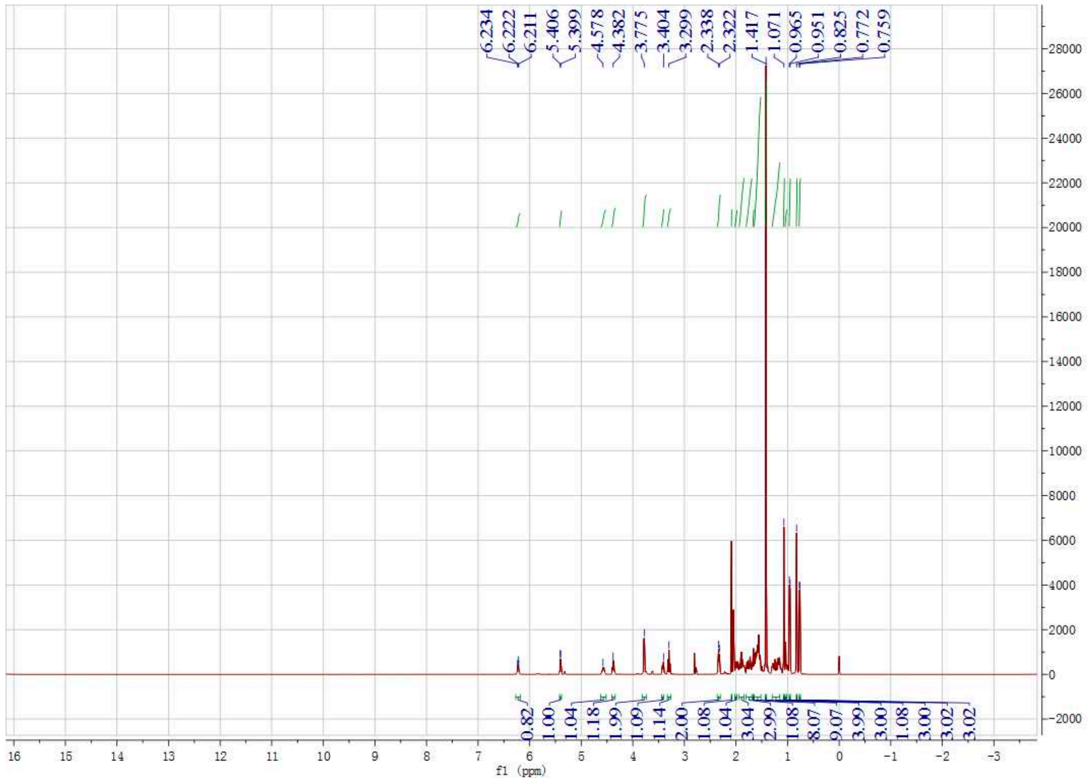

^{13}C NMR spectra of Compound DG-1

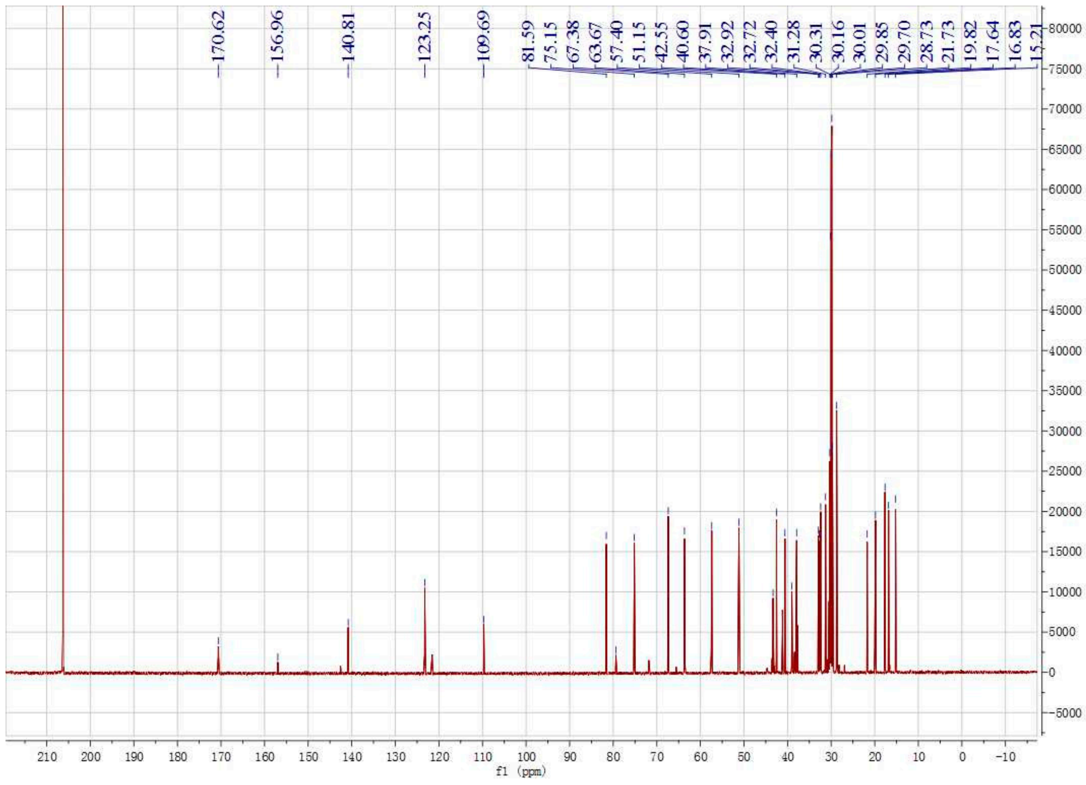

DG-2

^{1}H NMR spectra of Compound DG-2

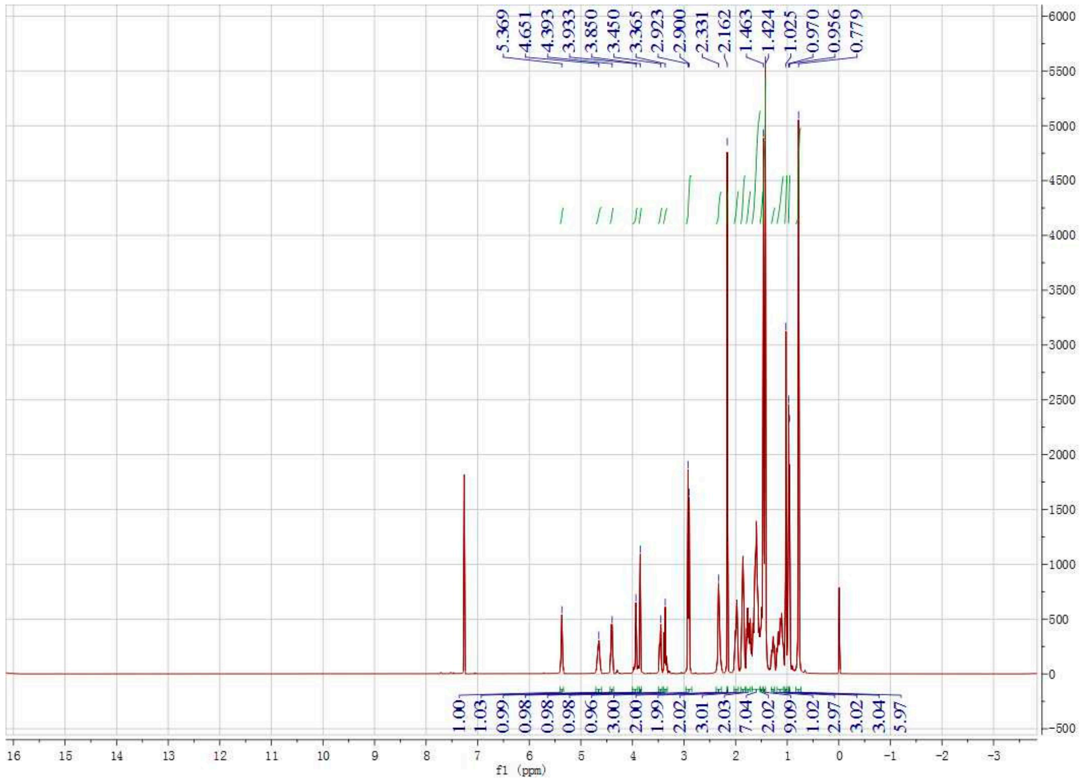

^{13}C NMR spectra of Compound DG-2

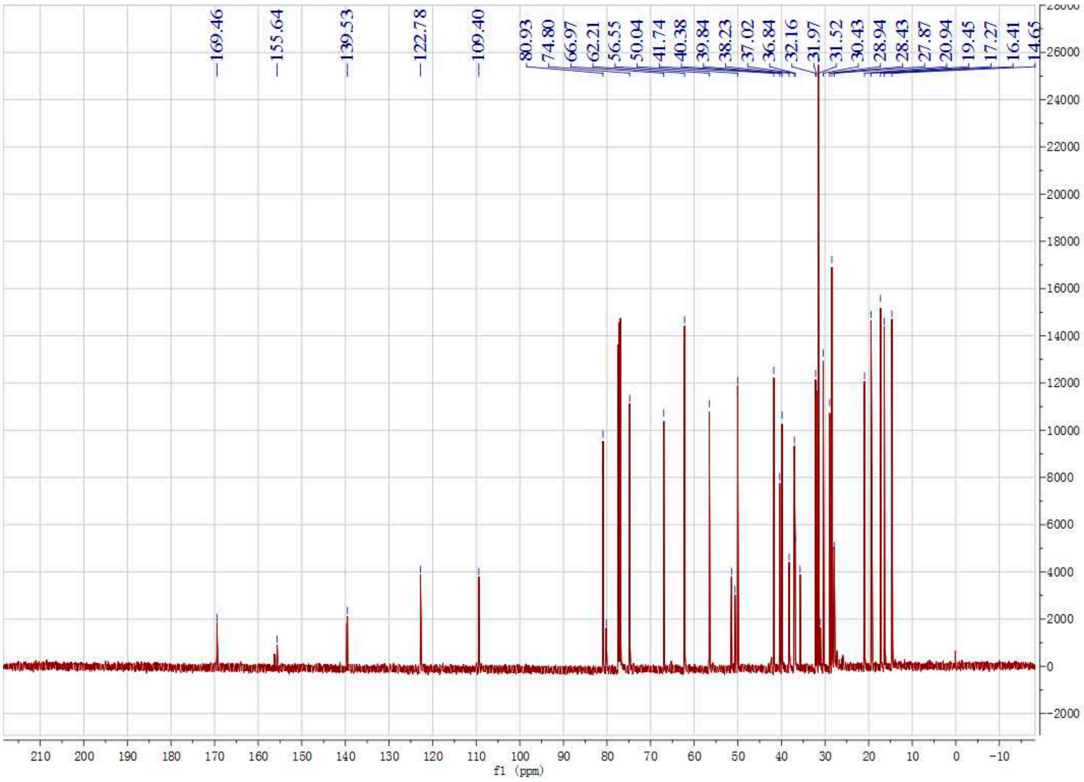

DG-3

^{1}H NMR spectra of Compound DG-3

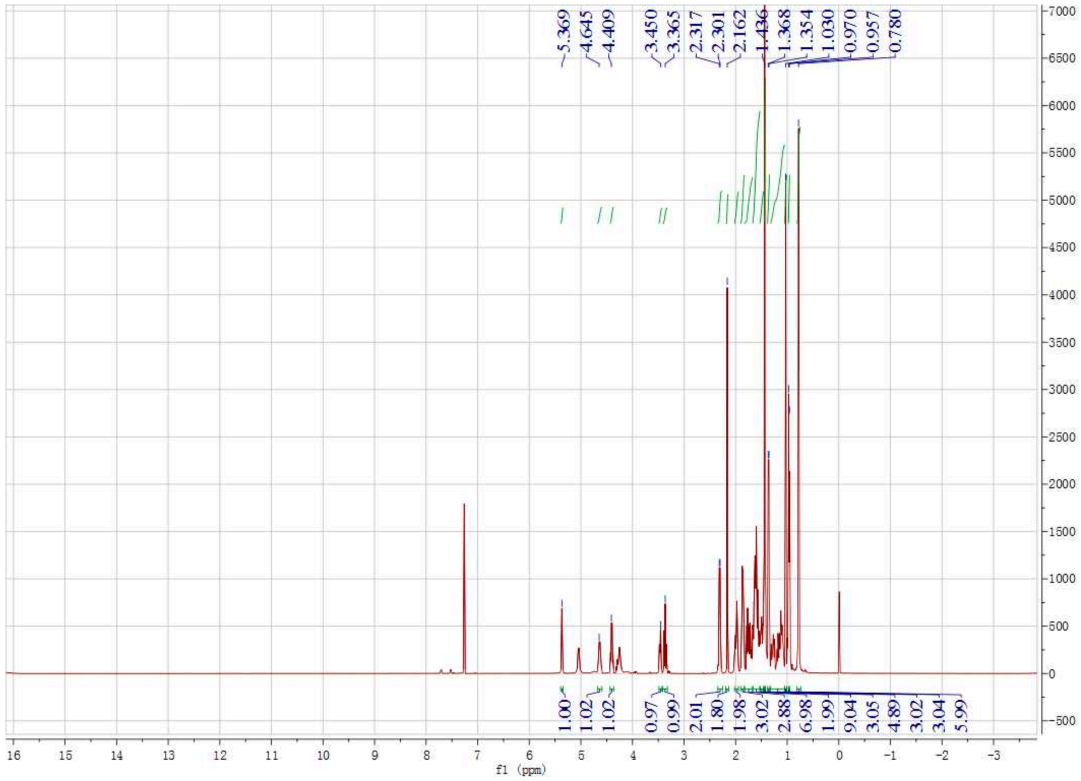

^{13}C NMR spectra of Compound DG-3

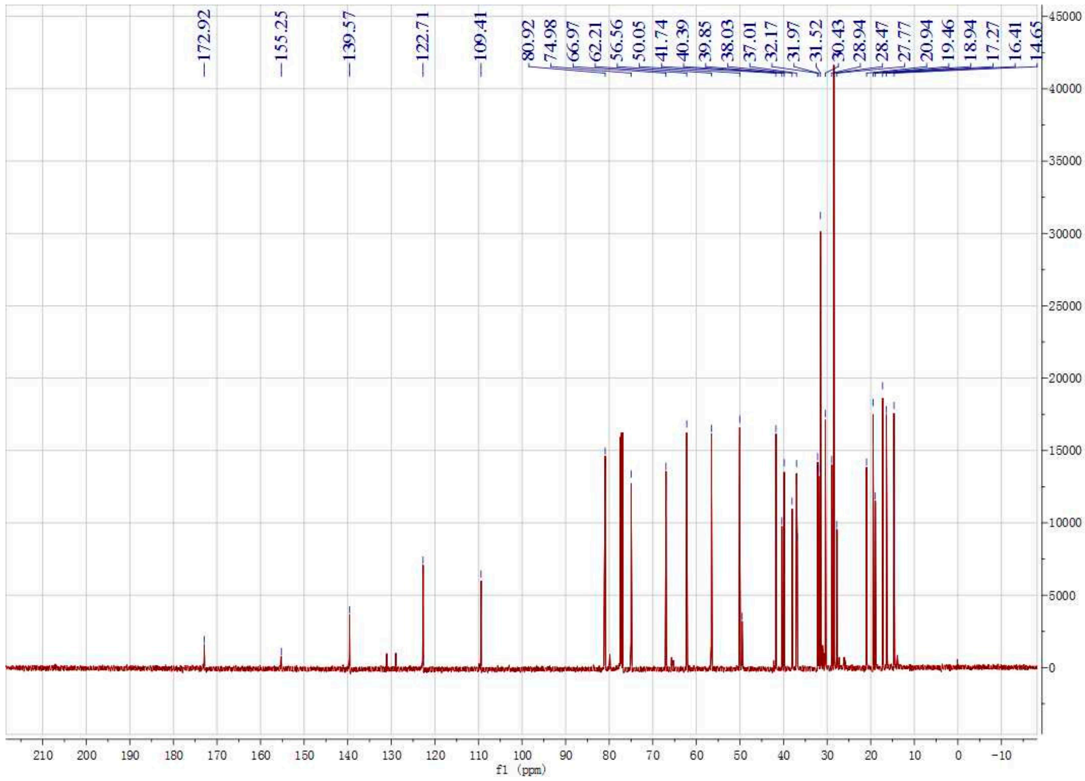

DG-4

^{1}H NMR spectra of Compound DG-4

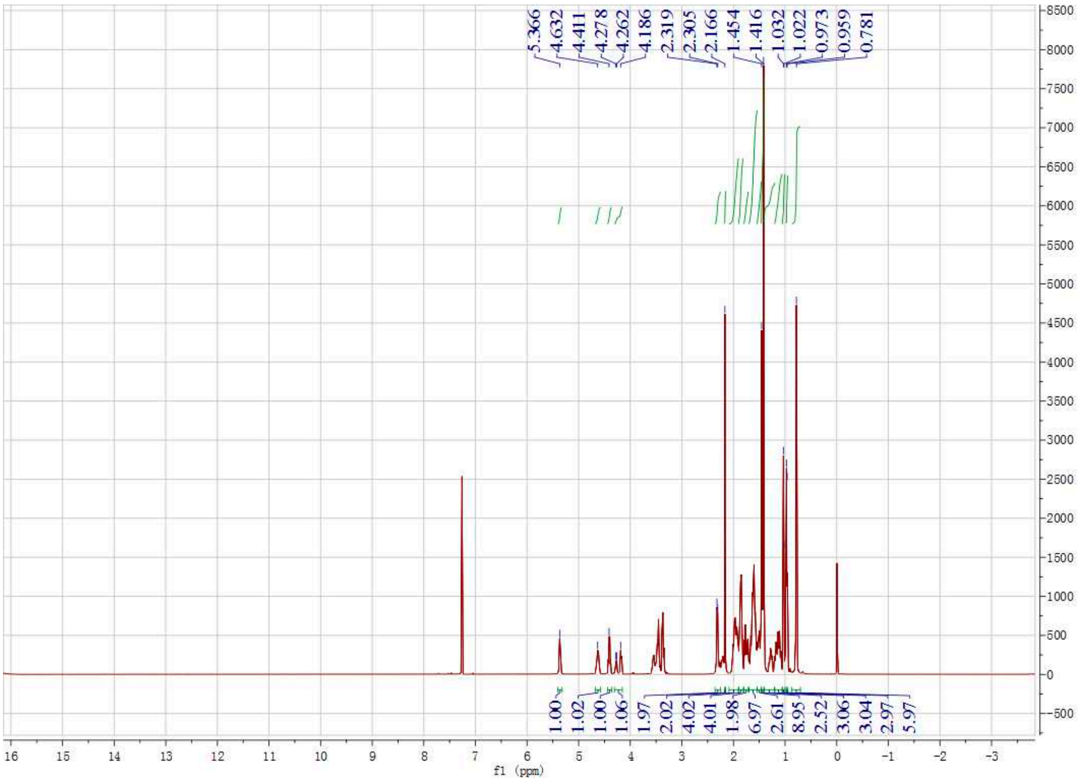

^{13}C NMR spectra of Compound DG-4

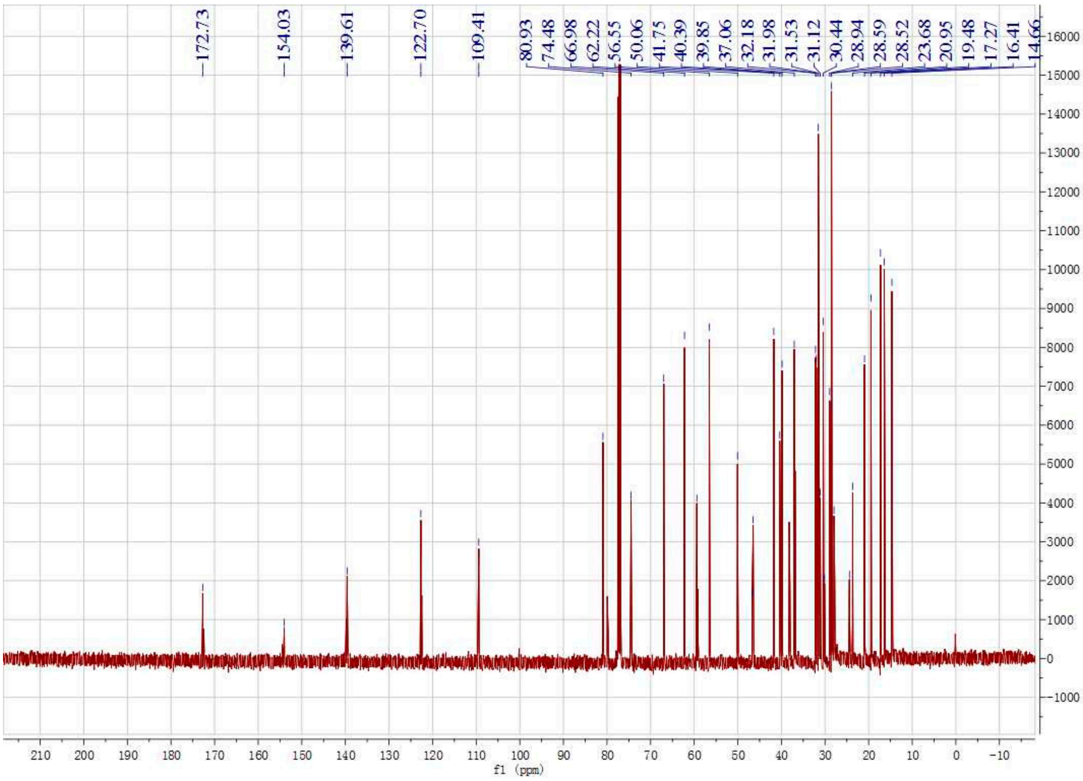

DG-5

^{1}H NMR spectra of Compound DG-5

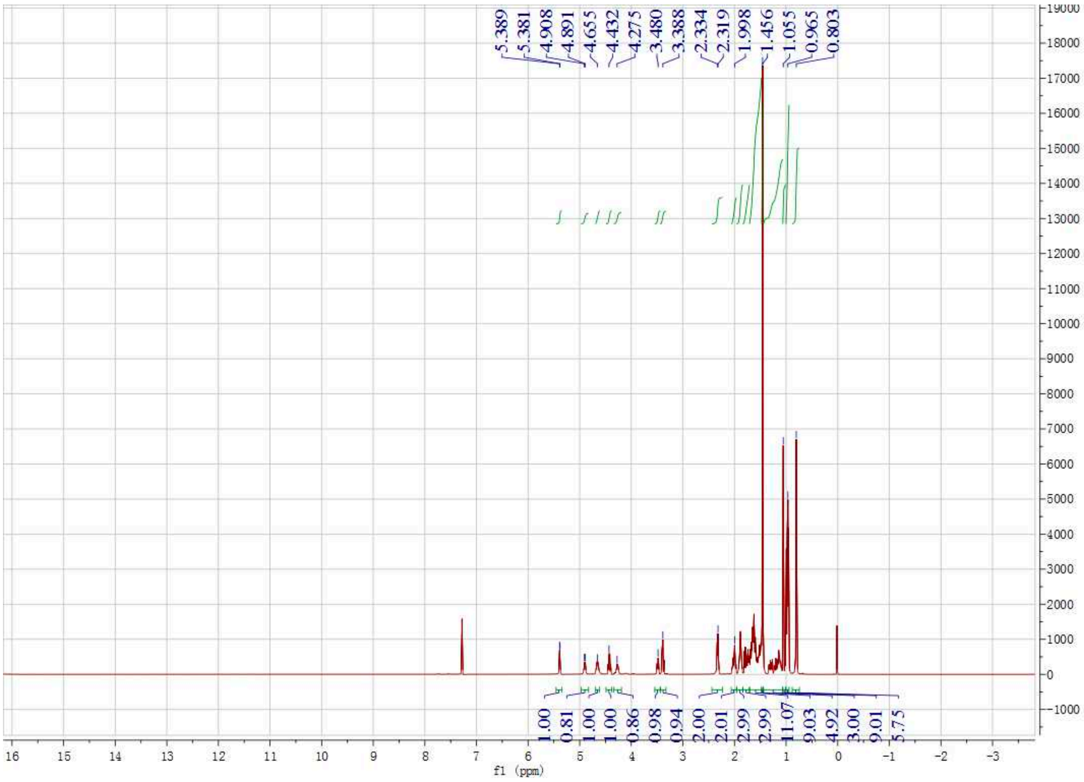

^{13}C NMR spectra of Compound DG-5

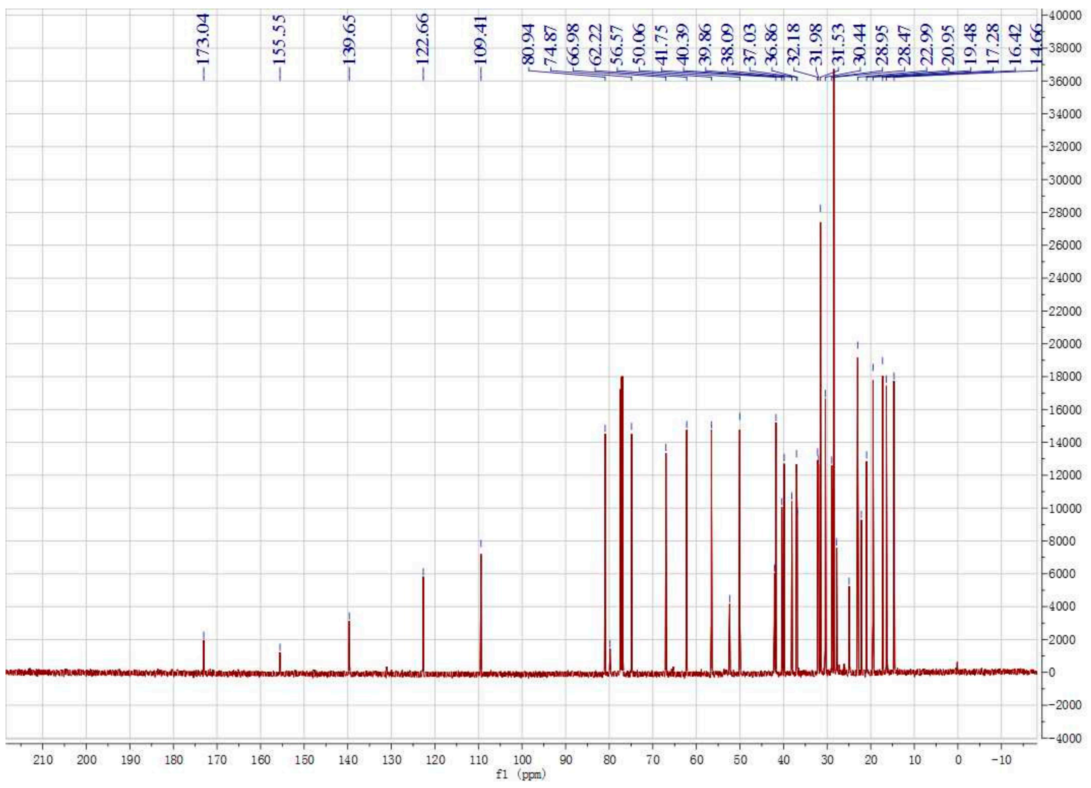

DG-6

^{1}H NMR spectra of Compound DG-6

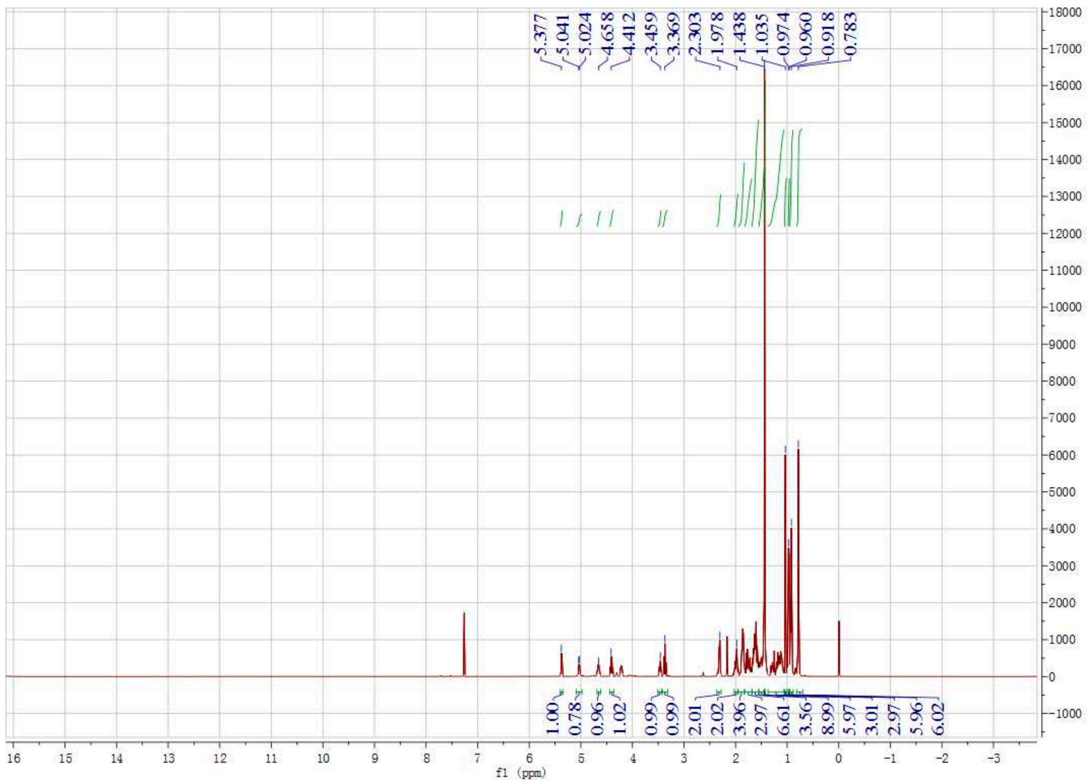

^{13}C NMR spectra of Compound DG-6

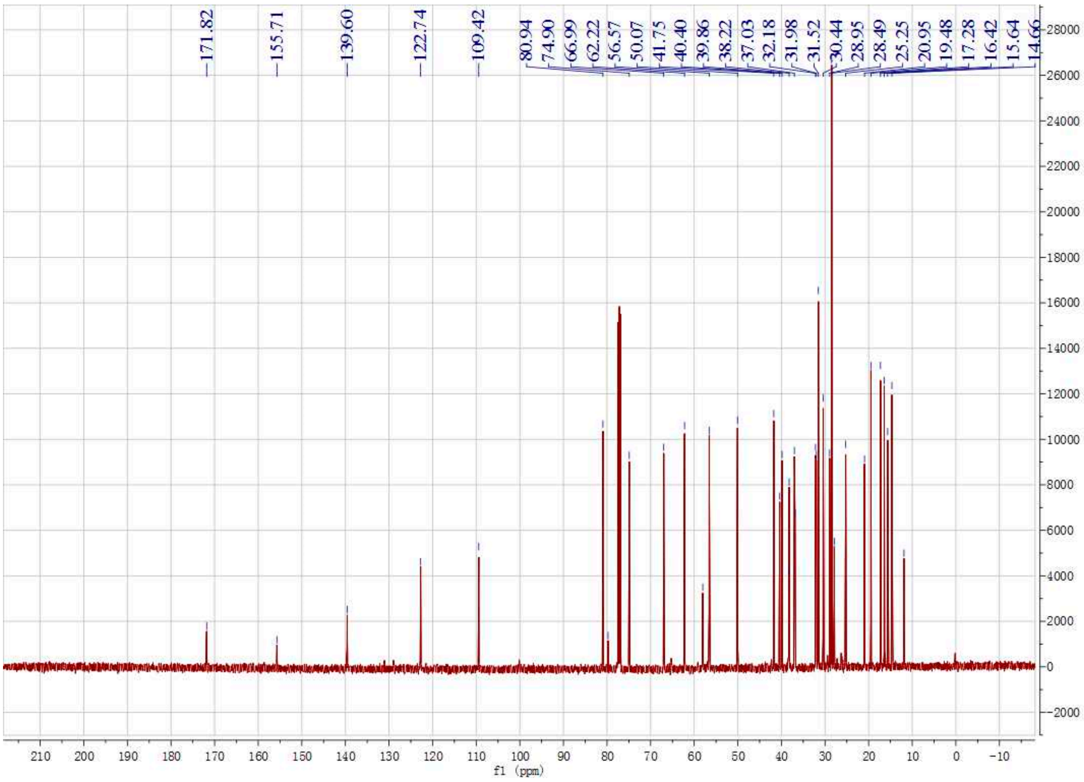

DG-7

^{1}H NMR spectra of Compound DG-7

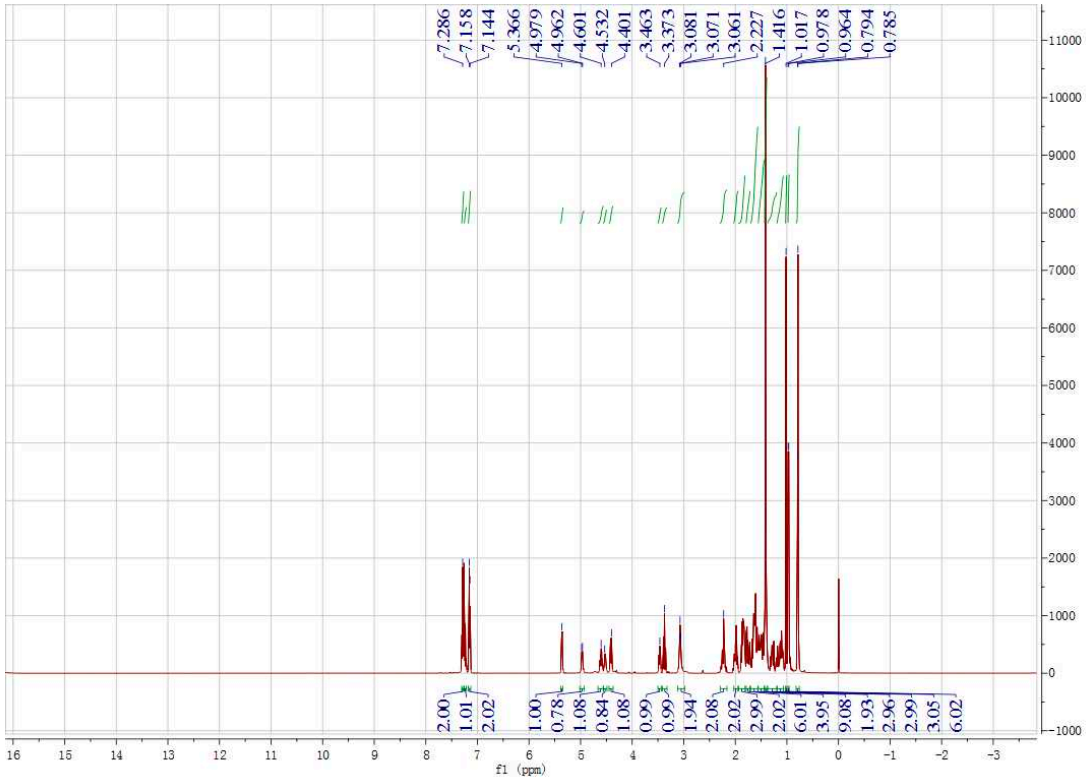

^{13}C NMR spectra of Compound DG-7

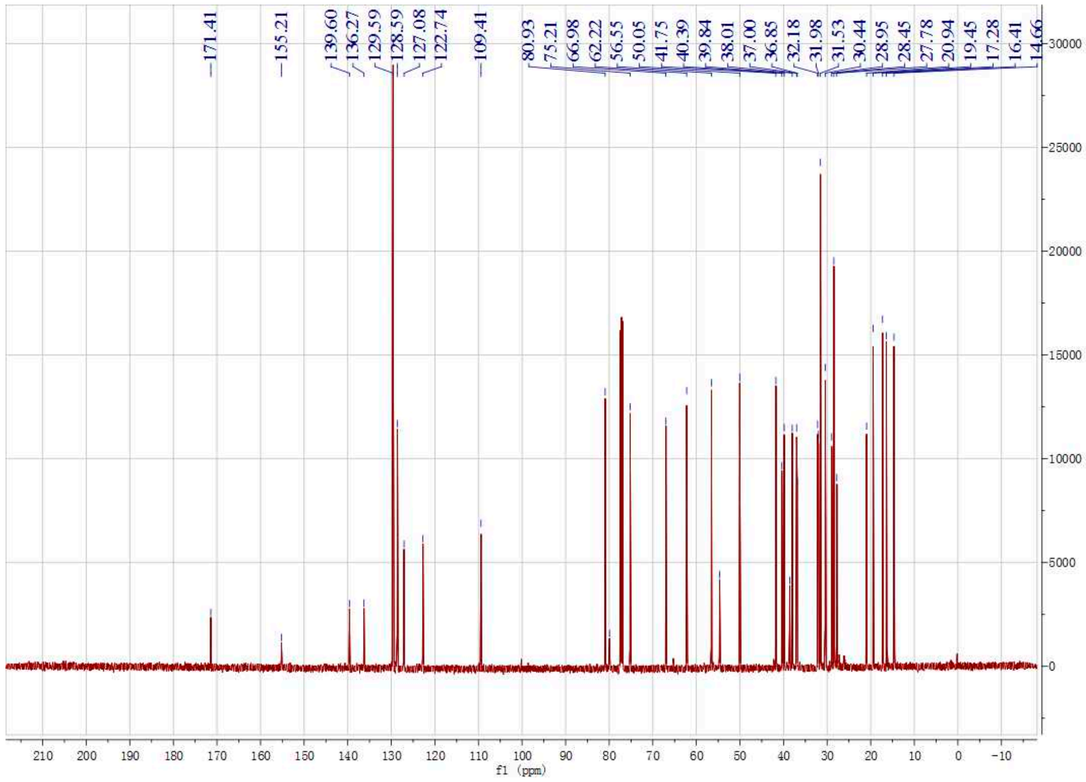

DG-8

^{1}H NMR spectra of Compound DG-8

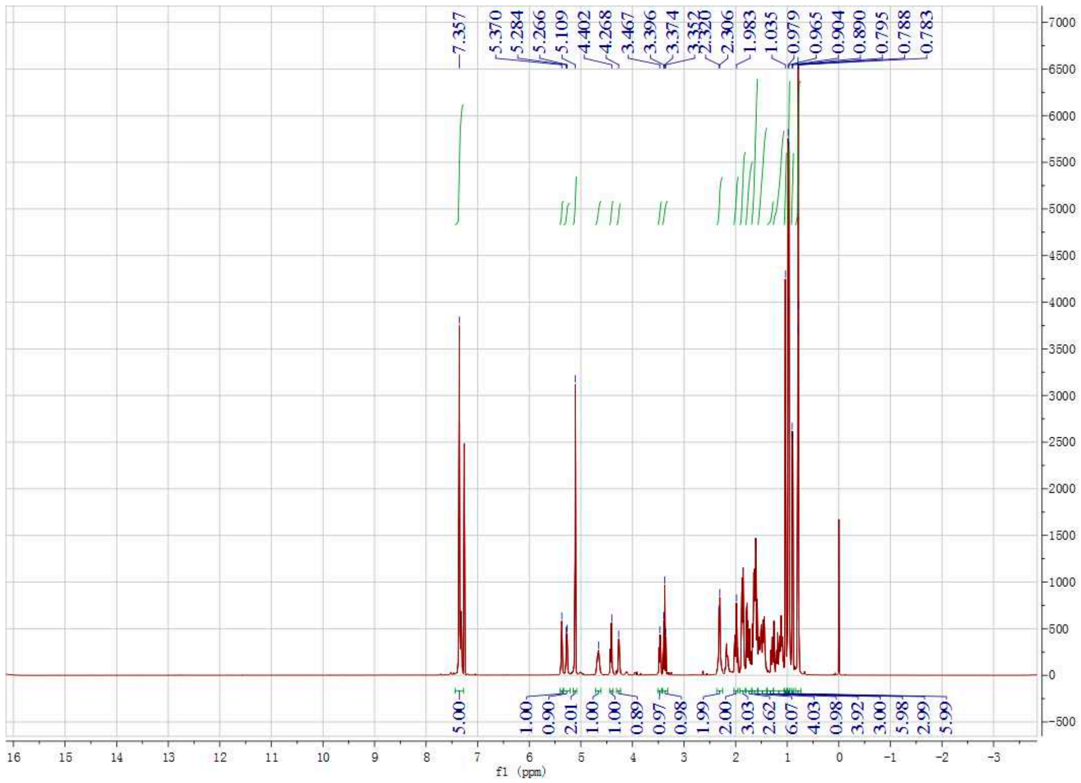

^{13}C NMR spectra of Compound DG-8

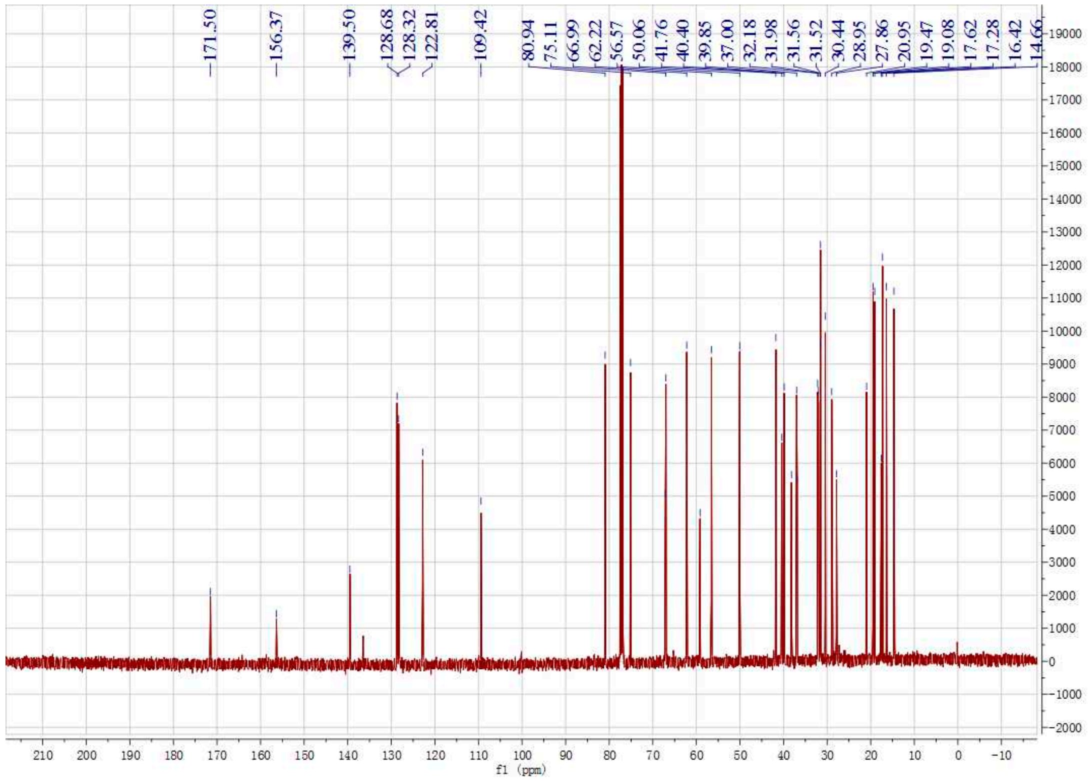

DG-9

^{1}H NMR spectra of Compound DG-9

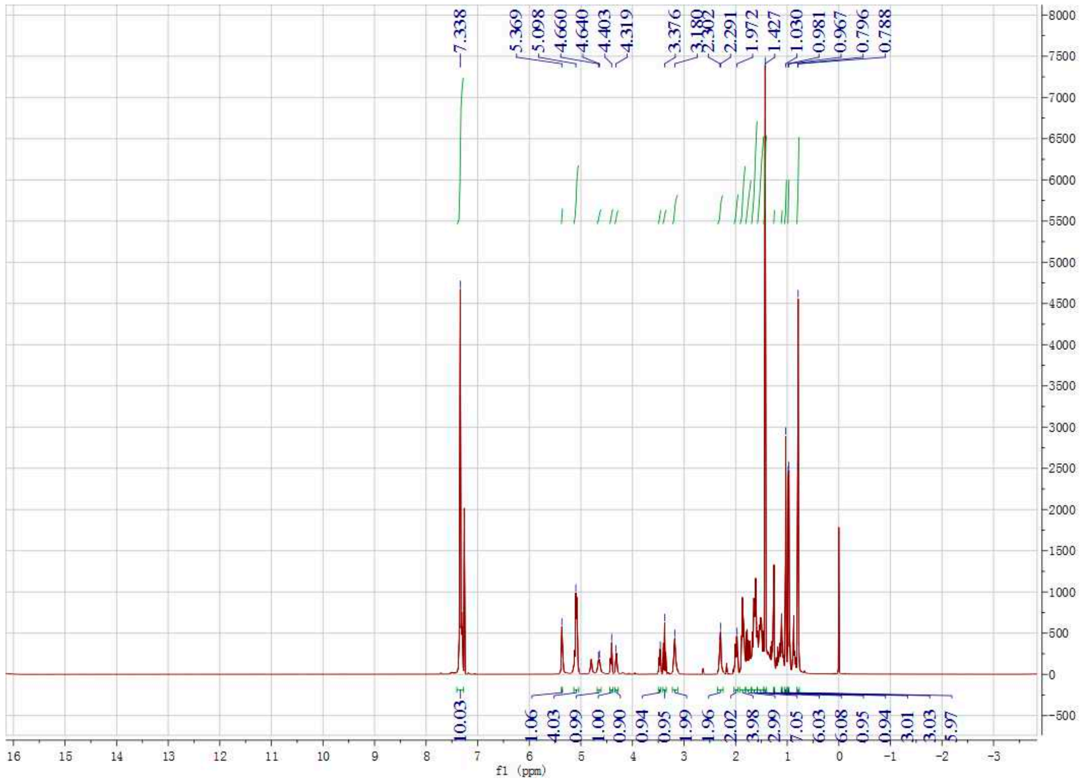

^{13}C NMR spectra of Compound DG-9

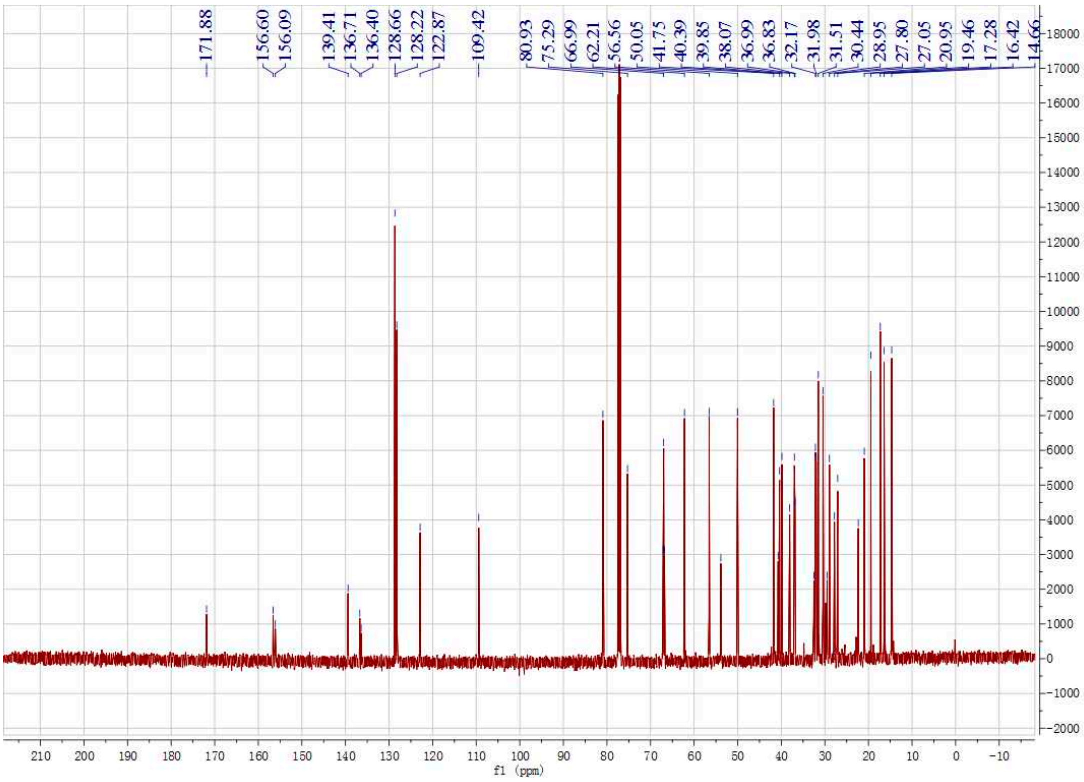

DG-10

^{1}H NMR spectra of Compound DG-10

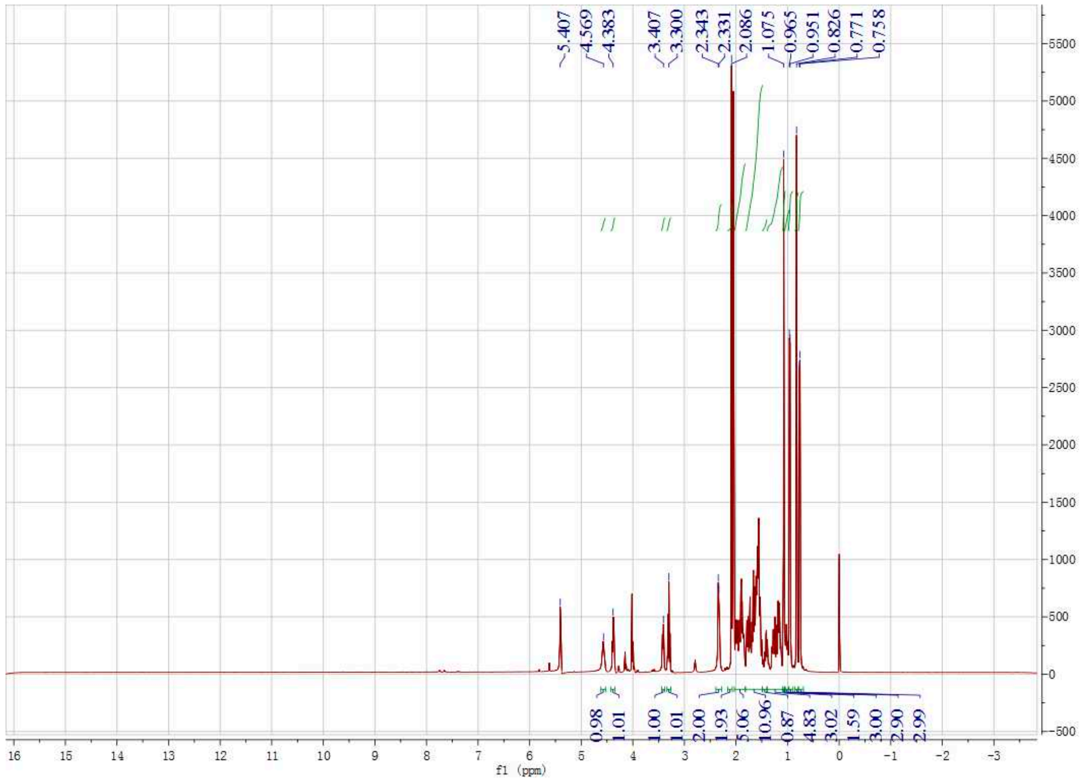

^{13}C NMR spectra of Compound DG-10

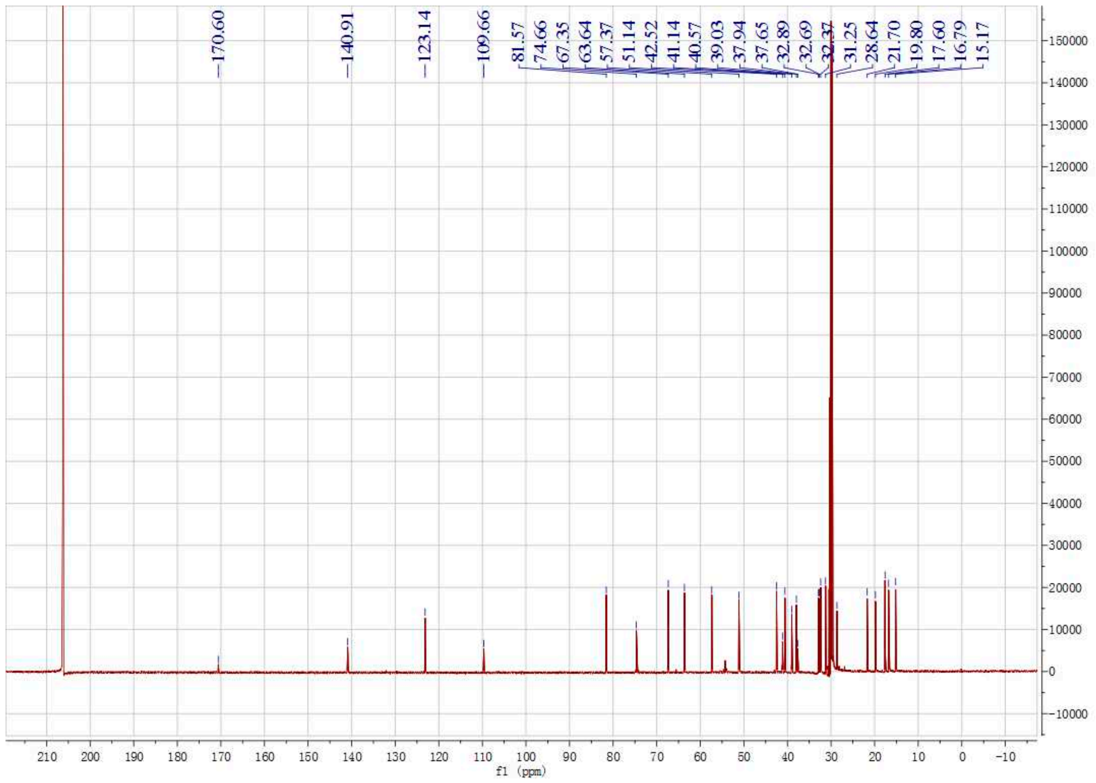

DG-11

^1H NMR spectra of Compound DG-11

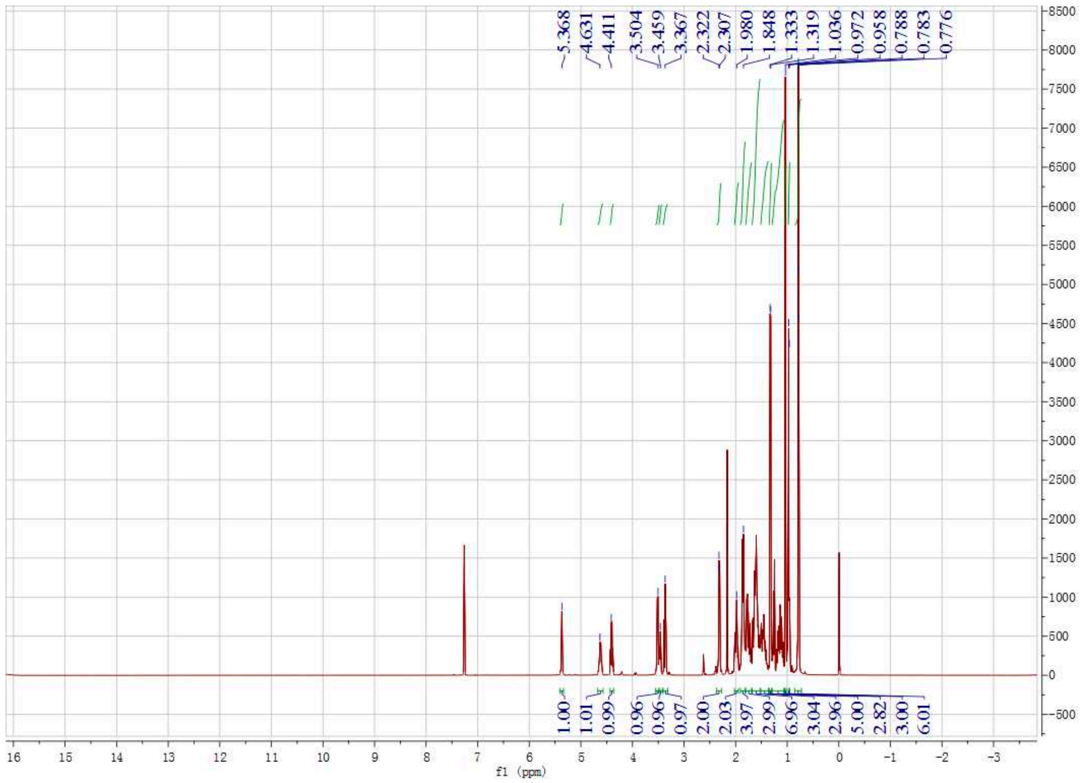

^{13}C NMR spectra of Compound DG-11

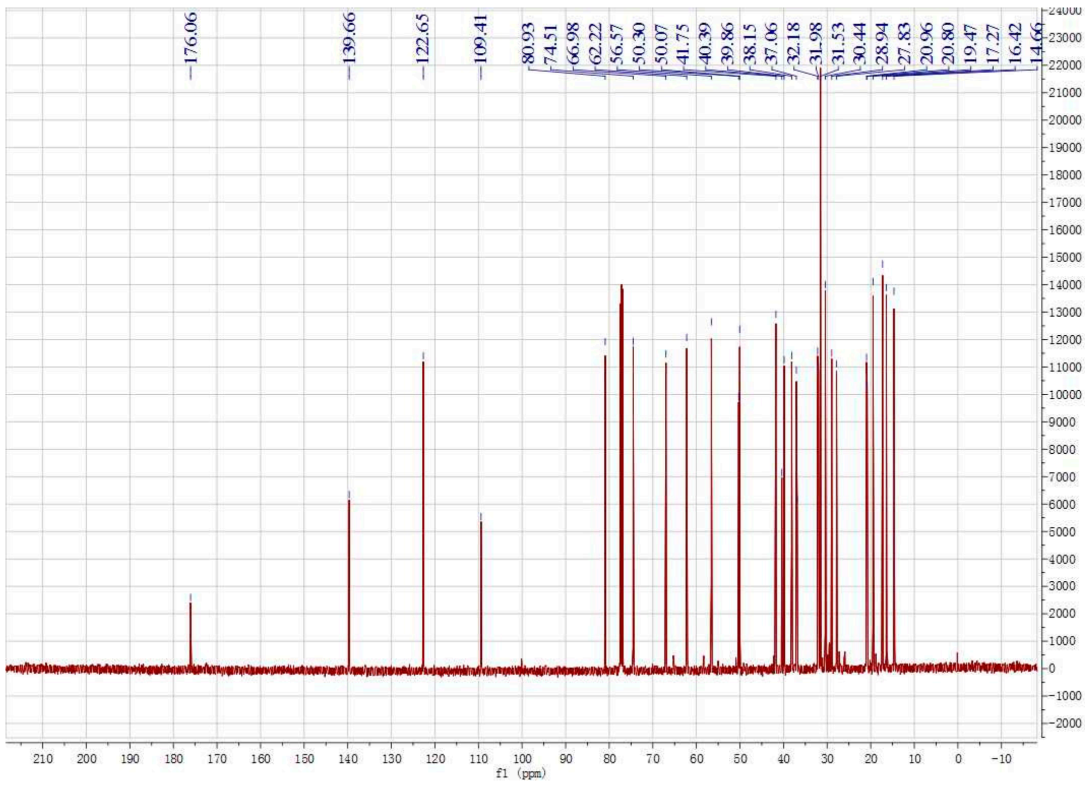

DG-12

^{1}H NMR spectra of Compound DG-12

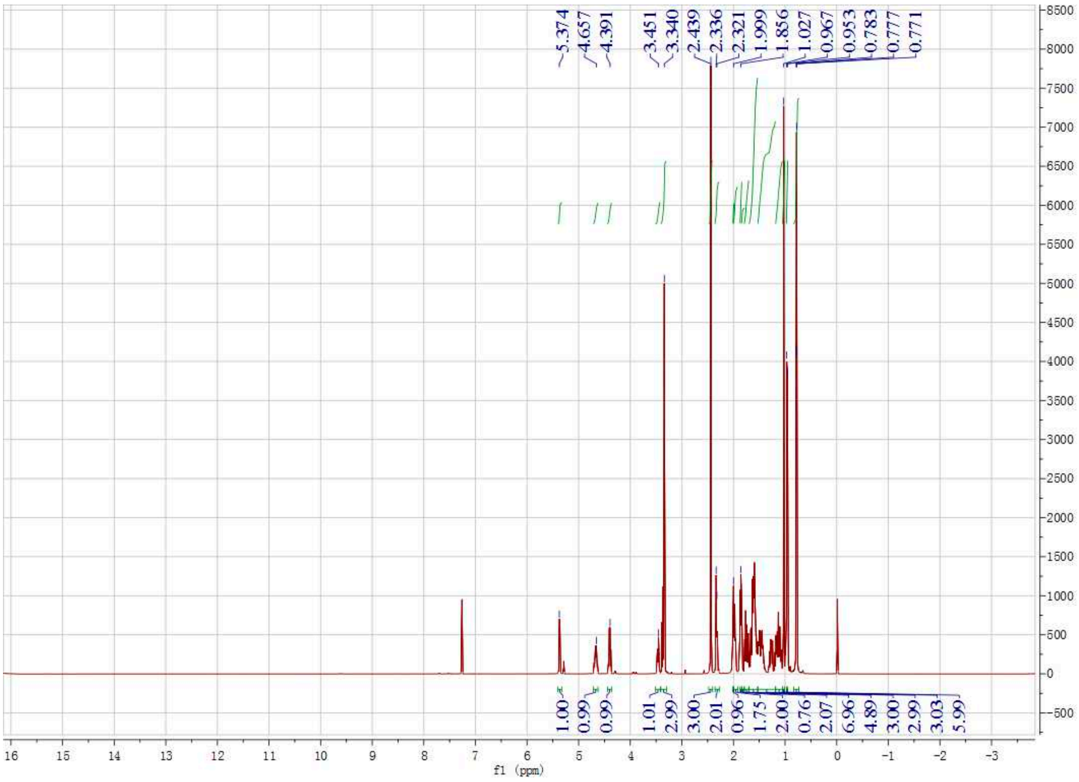

^{13}C NMR spectra of Compound DG-12

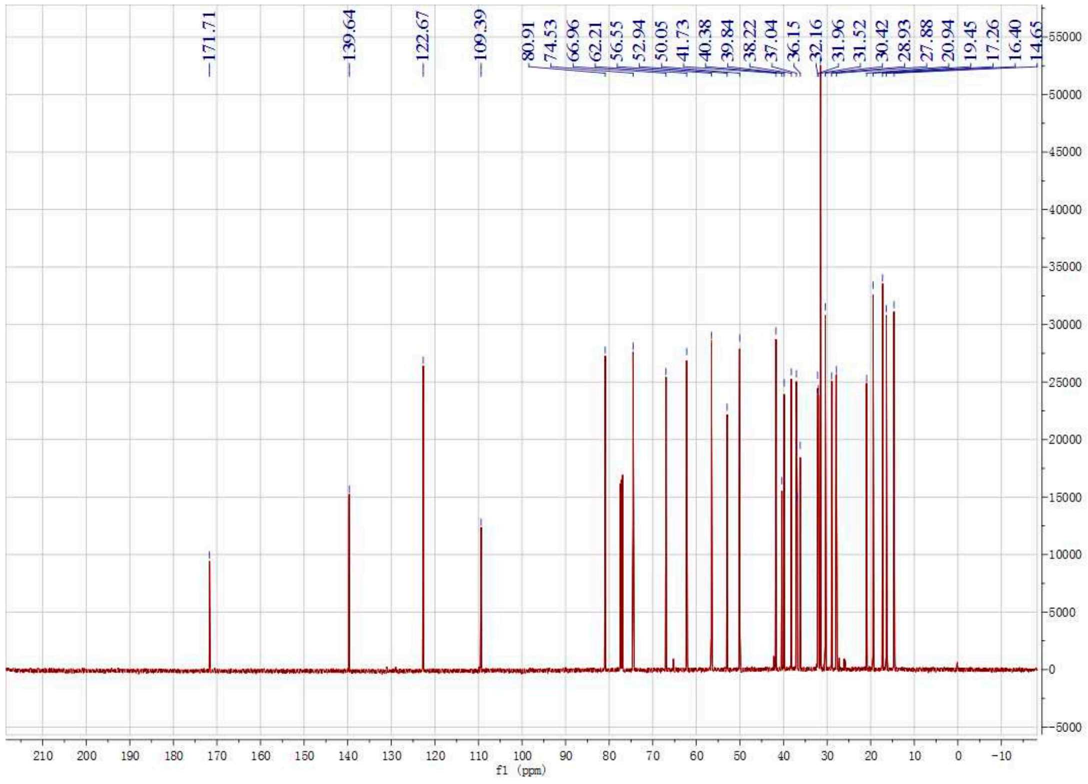

DG-13

^{1}H NMR spectra of Compound DG-13

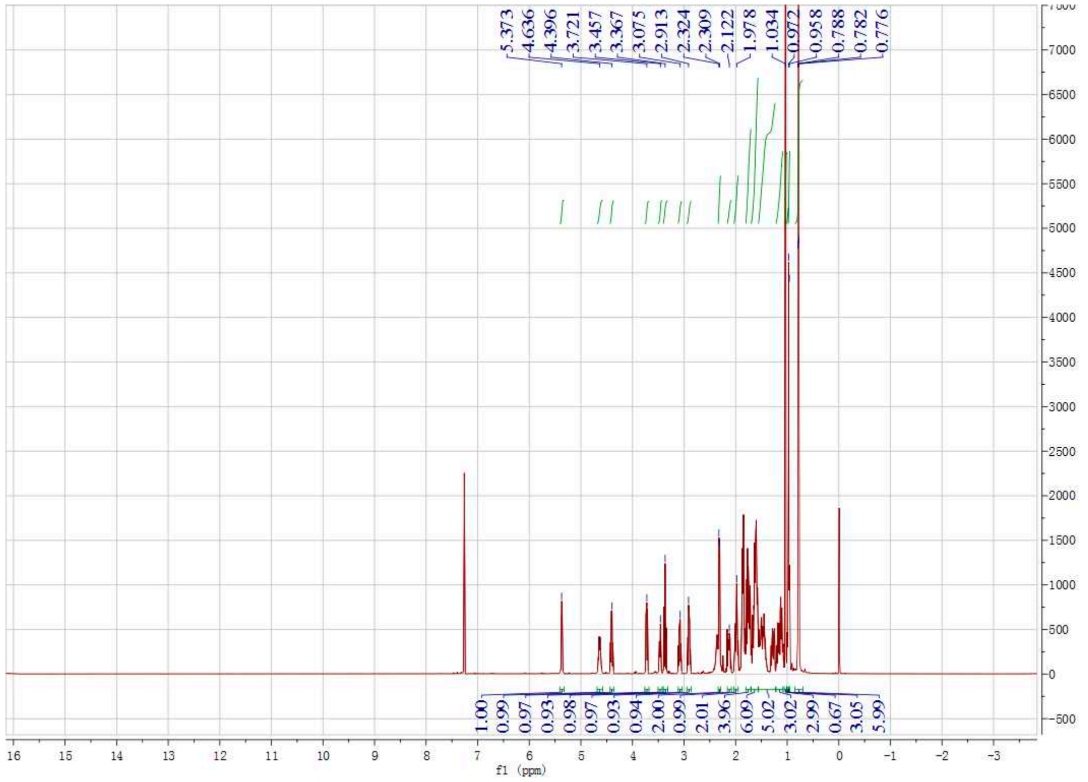

^{13}C NMR spectra of Compound DG-13

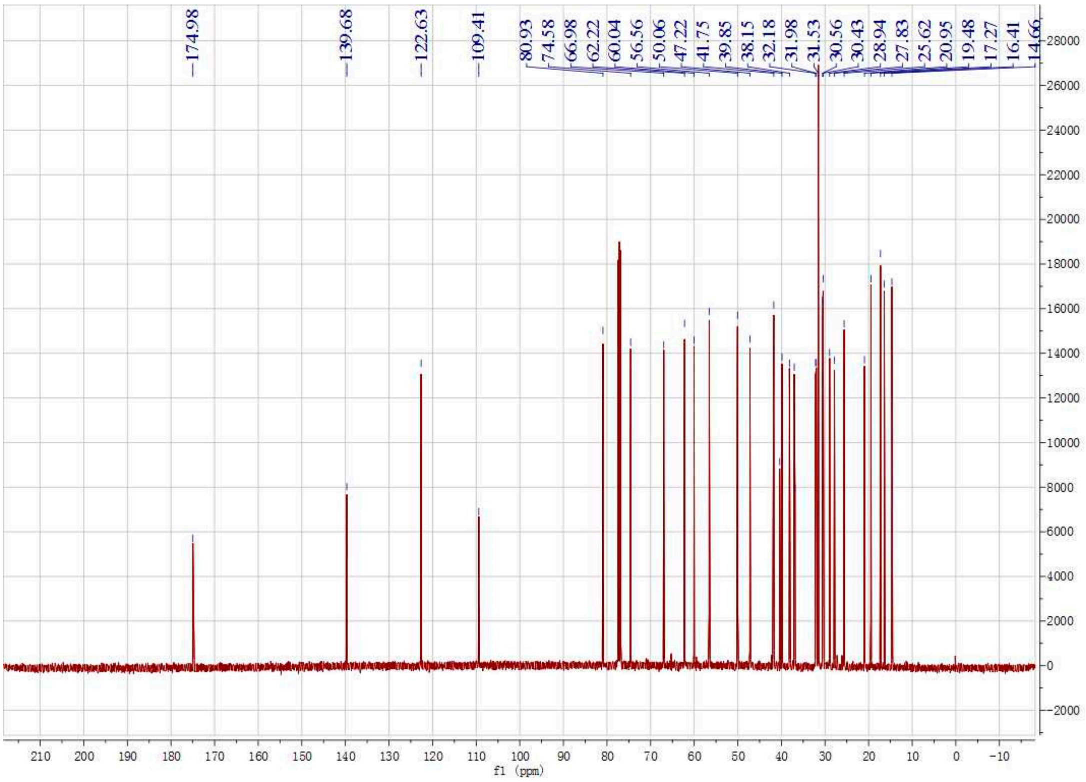

DG-14

^{1}H NMR spectra of Compound DG-14

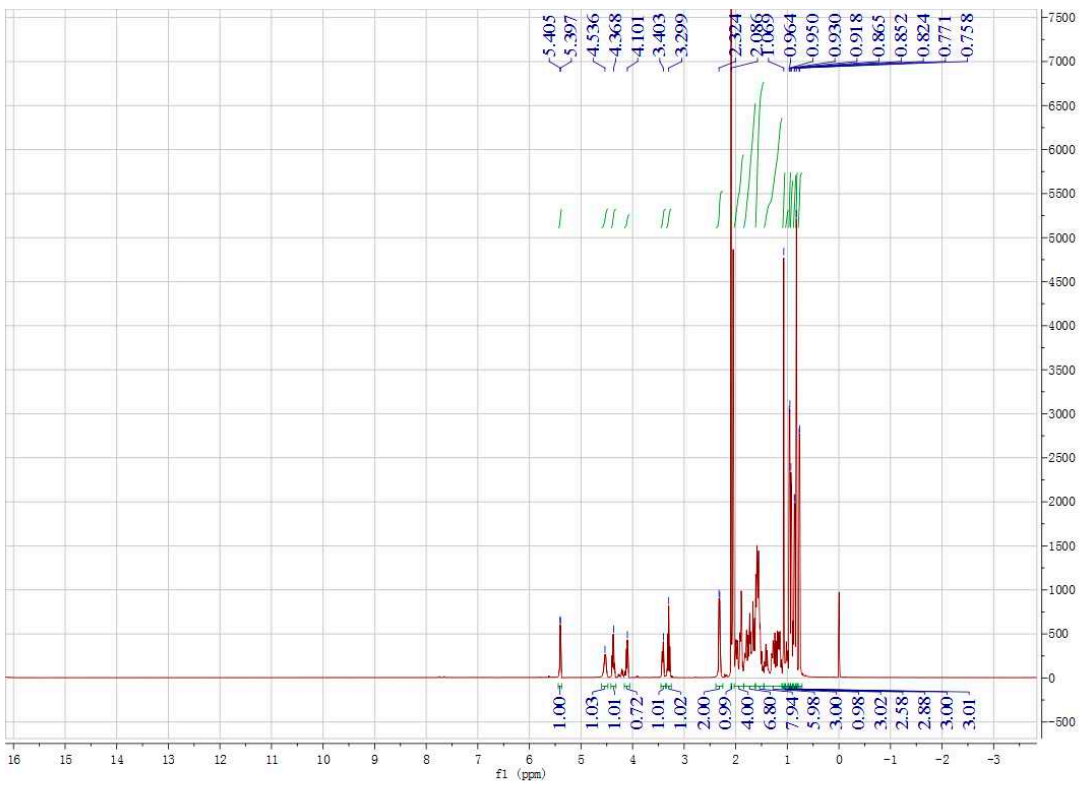

^{13}C NMR spectra of Compound DG-14

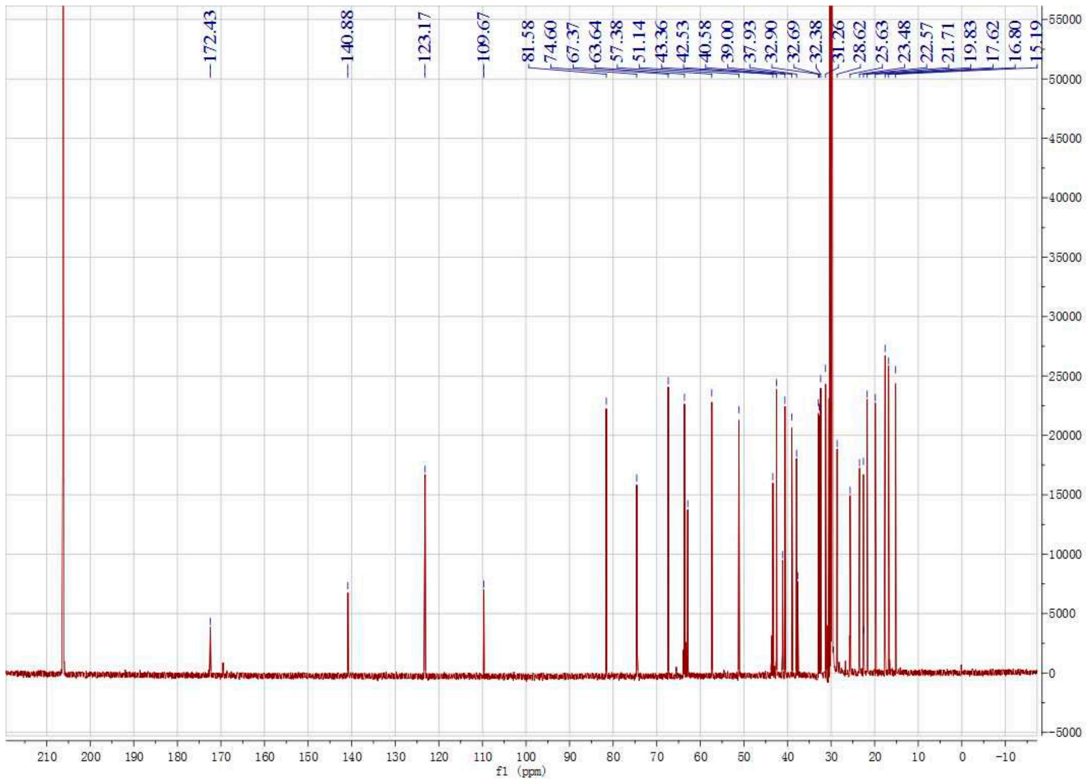

DG-15

^{1}H NMR spectra of Compound DG-15

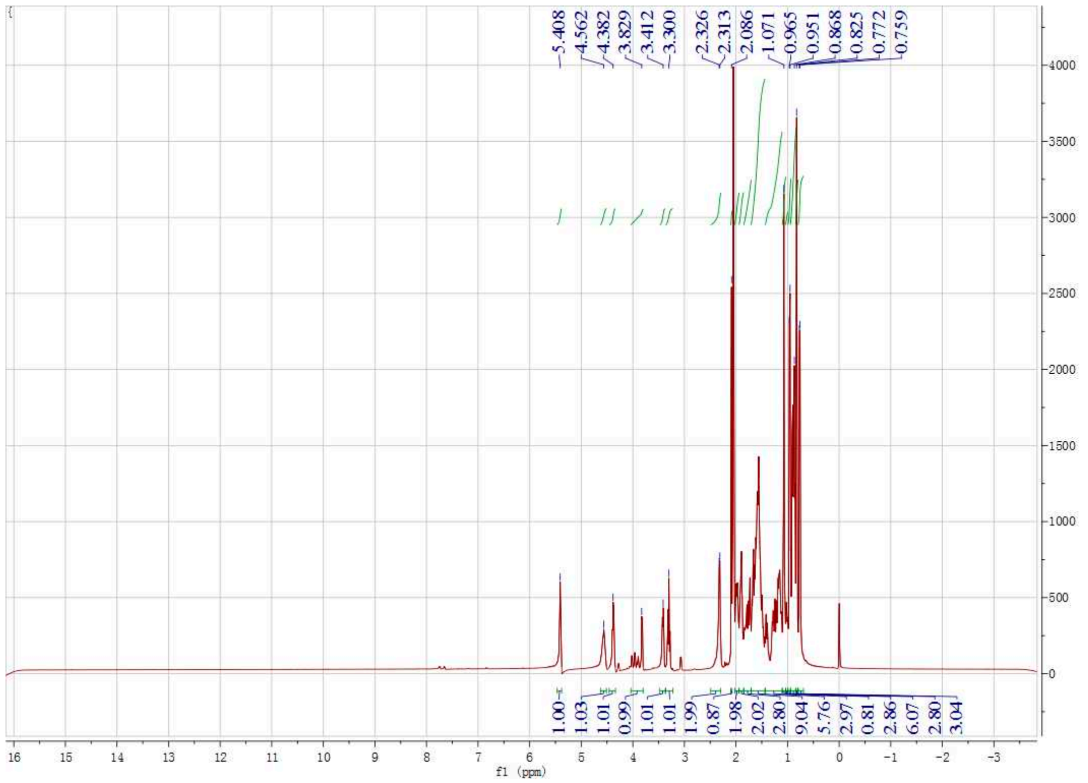

^{13}C NMR spectra of Compound DG-15

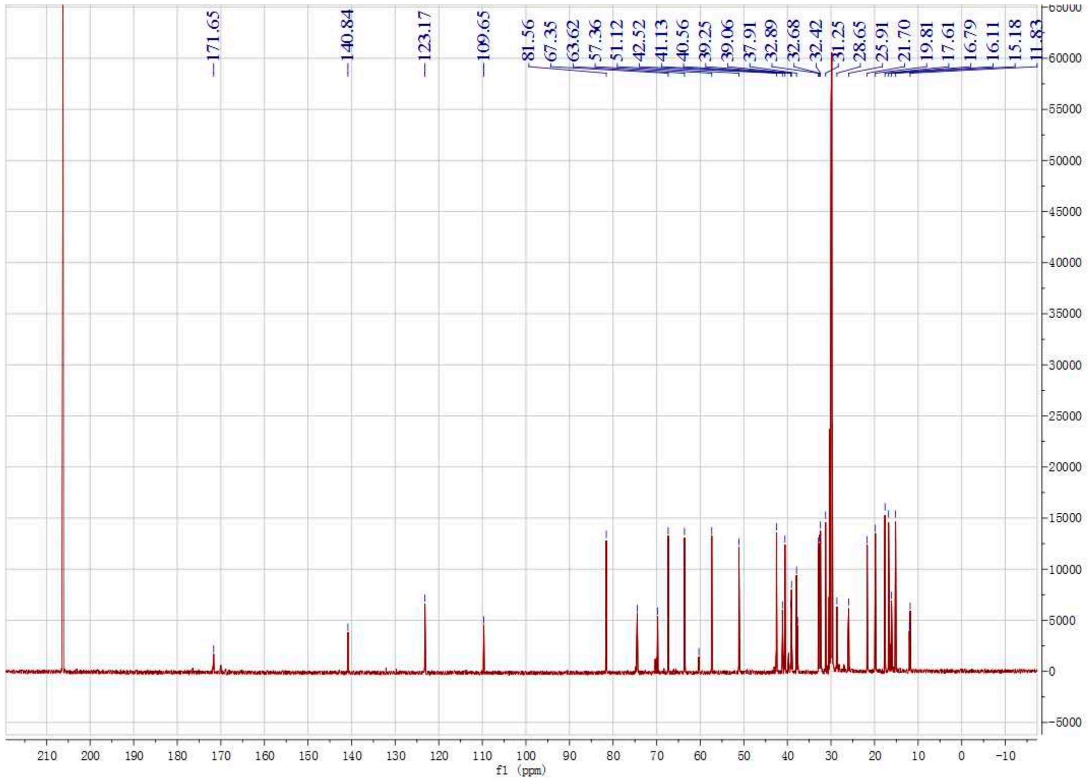

DG-16

^{1}H NMR spectra of Compound DG-16

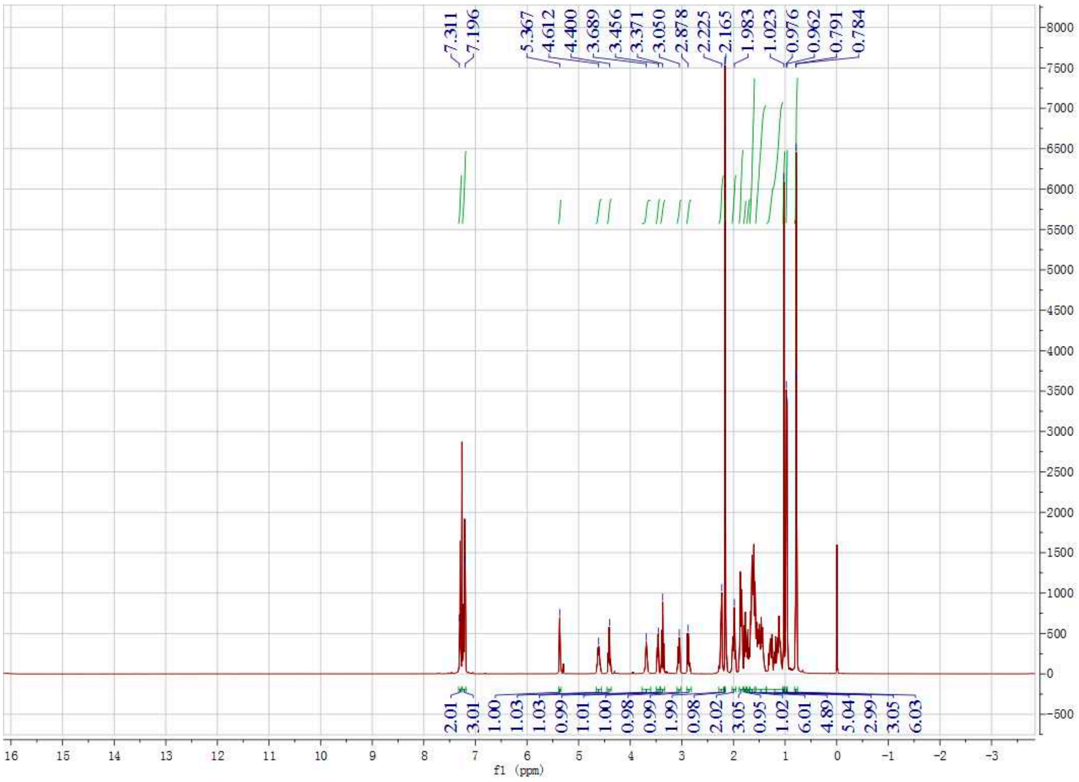

^{13}C NMR spectra of Compound DG-16

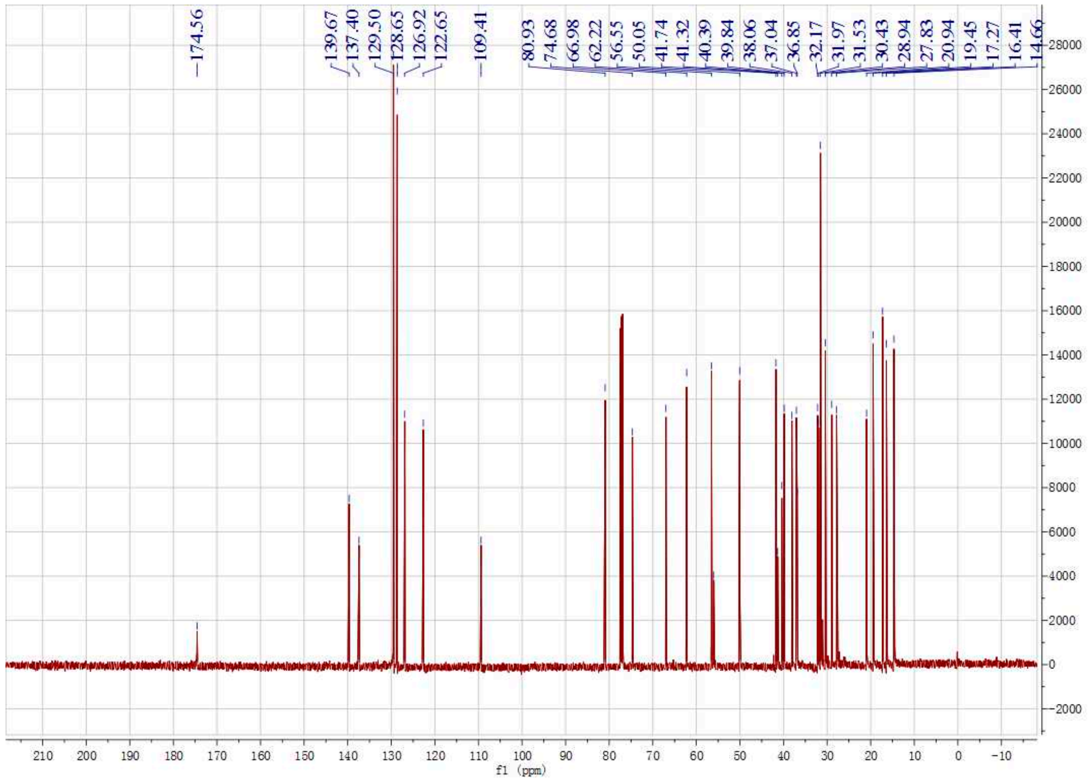

DG-17

^{1}H NMR spectra of Compound DG-17

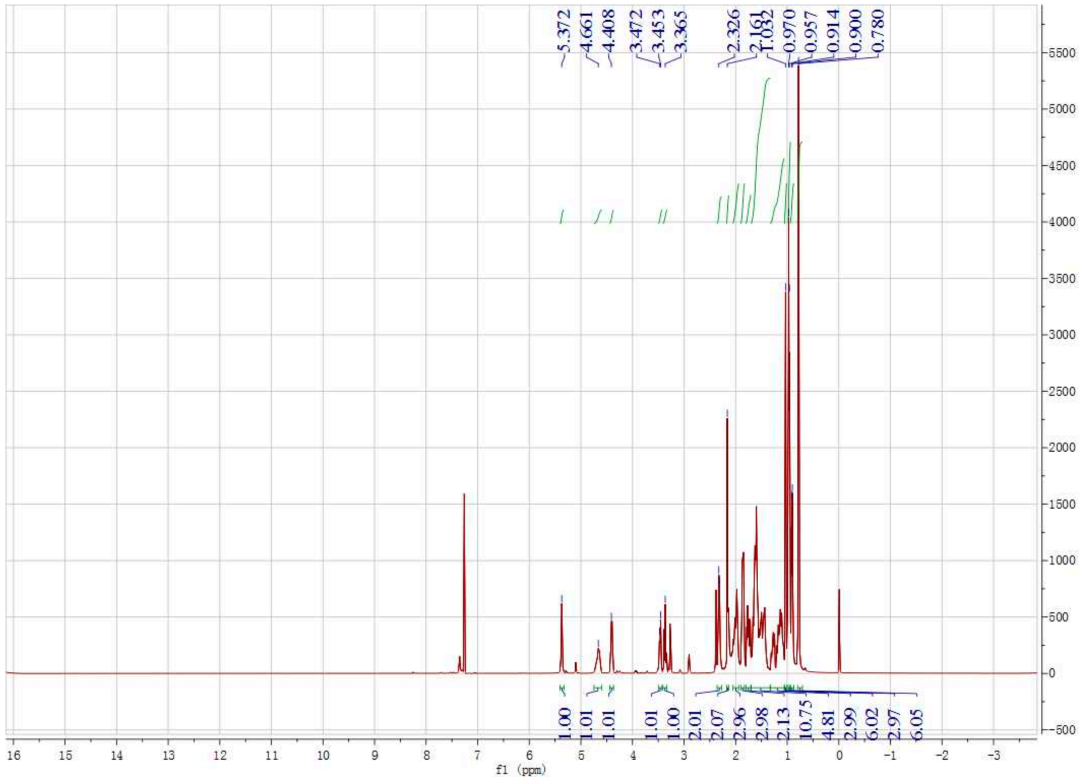

^{13}C NMR spectra of Compound DG-17

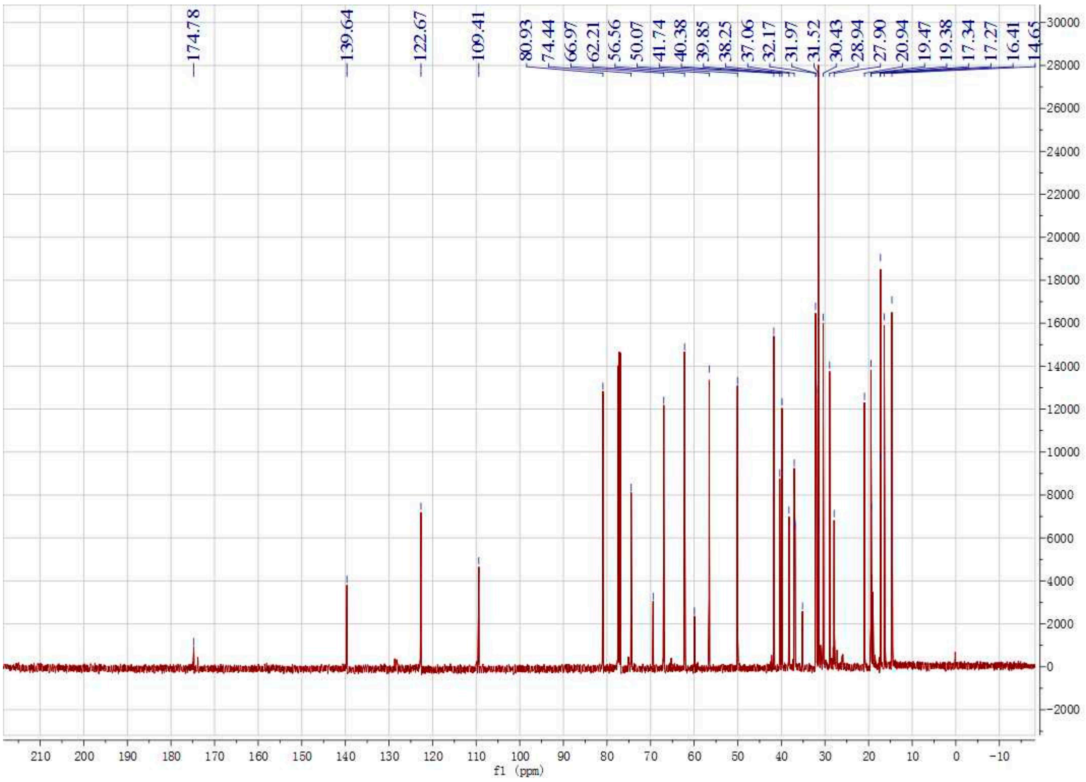

DG-18

^{1}H NMR spectra of Compound DG-18

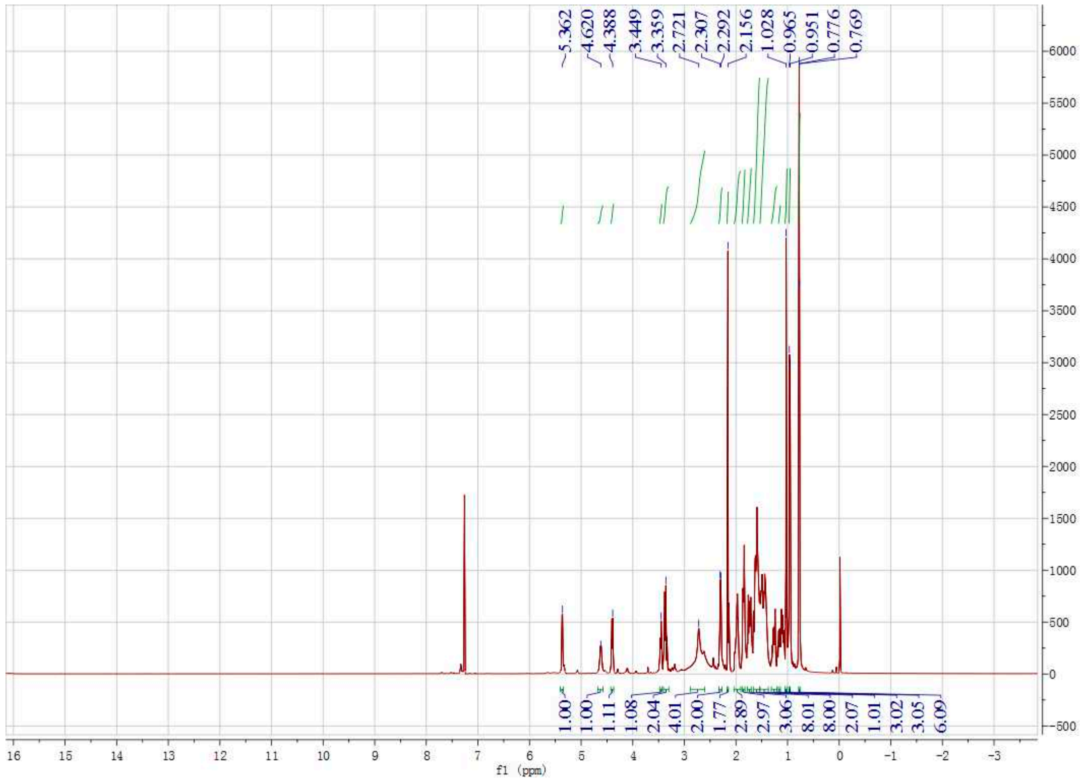

^{13}C NMR spectra of Compound DG-18

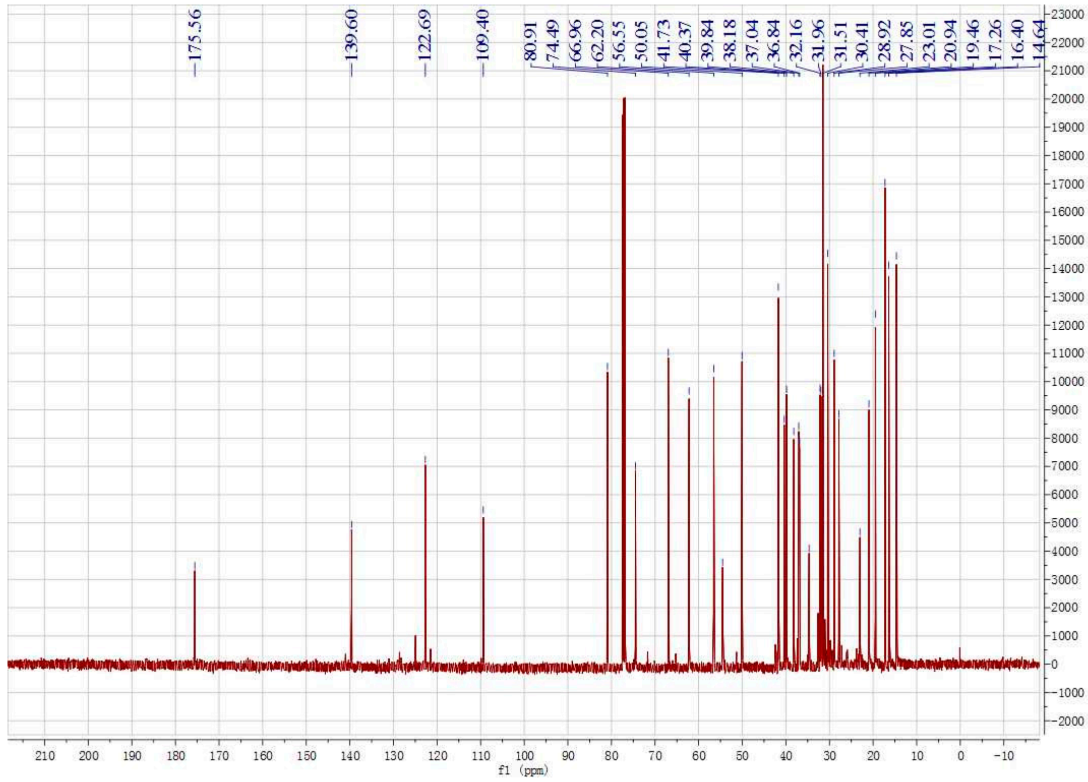

Supplement: Supplementary file 1 [file molecules-24-04025-s001.pdf]
